# Psycho-Neuro-Endocrine-Immunological Basis of the Placebo Effect: Potential Applications beyond Pain Therapy

**DOI:** 10.3390/ijms23084196

**Published:** 2022-04-11

**Authors:** Ángel Ortega, Juan Salazar, Néstor Galban, Milagros Rojas, Daniela Ariza, Mervin Chávez-Castillo, Manuel Nava, Manuel E. Riaño-Garzón, Edgar Alexis Díaz-Camargo, Oscar Medina-Ortiz, Valmore Bermúdez

**Affiliations:** 1 Endocrine and Metabolic Diseases Research Center, School of Medicine, University of Zulia, Maracaibo 4004, Venezuela; angelort94@gmail.com (Á.O.); juanjsv18@hotmail.com (J.S.); nestorag17@gmail.com (N.G.); migarocafi@gmail.com (M.R.); arizathings@gmail.com (D.A.); mervinch12@gmail.com (M.C.-C.); manuelnava93@gmail.com (M.N.); 2 Facultad de Ciencias Jurídicas y Sociales, Universidad Simón Bolívar, Cúcuta 540006, Colombia; m.riano@unisimonbolivar.edu.co (M.E.R.-G.); e.diaz@unisimonbolivar.edu.co (E.A.D.-C.); 3 Facultad de Medicina, Universidad de Santander, Cúcuta 540003, Colombia; o.medina@unisimonbolivar.edu.co; 4 Universidad Simón Bolívar, Facultad de Ciencias de la Salud, Barranquilla 080002, Colombia

**Keywords:** placebo effect, psychoneuroimmunology, conditioning, cannabinoids, opioid, hormones, analgesia, pain, depression, Parkinson’s disease

## Abstract

The placebo effect can be defined as the improvement of symptoms in a patient after the administration of an innocuous substance in a context that induces expectations regarding its effects. During recent years, it has been discovered that the placebo response not only has neurobiological functions on analgesia, but that it is also capable of generating effects on the immune and endocrine systems. The possible integration of changes in different systems of the organism could favor the well-being of the individuals and go hand in hand with conventional treatment for multiple diseases. In this sense, classic conditioning and setting expectations stand out as psychological mechanisms implicated in the placebo effect. Recent advances in neuroimaging studies suggest a relationship between the placebo response and the opioid, cannabinoid, and monoaminergic systems. Likewise, a possible immune response conditioned by the placebo effect has been reported. There is evidence of immune suppression conditioned through the insular cortex and the amygdala, with noradrenalin as the responsible neurotransmitter. Finally, a conditioned response in the secretion of different hormones has been determined in different studies; however, the molecular mechanisms involved are not entirely known. Beyond studies about its mechanism of action, the placebo effect has proved to be useful in the clinical setting with promising results in the management of neurological, psychiatric, and immunologic disorders. However, more research is needed to better characterize its potential use. This review integrates current knowledge about the psycho-neuro-endocrine-immune basis of the placebo effect and its possible clinical applications.

## 1. Introduction

In ancient times, diseases were attributed to divine punishments, and priests or shamans would have the function of both doctors and spiritual guides. Therefore, if there was any improvement in the health of their patients, it was not due to medicinal principles but due to “rituals”, which are now recognized as the placebo effect [1]. The first time the word placebo (from the Latin word placēbō, complacere) was used in the medical setting was in a series of conferences dictated in 1772 by British doctor William Cullen. Since then, the meaning of this word has evolved, and it is currently defined as a substance without an active principle given to patients to improve their health or used in clinical trials to compare the performance of a new drug [1,2].

The placebo effect can be defined as any improvement in health caused by an intervention that has no direct physical-chemical effect on the ailment [3]. In modern medicine, a placebo has been widely used as a point of comparison in clinical trials, but it was not until the last century that the placebo effect was observed as a phenomenon in and of itself [4].

The veracity of the placebo effect was a controversial concept. Furthermore, it was thought that if an ailment improved as a result of an inactive intervention, the ailment must have been false from the start. However, research has demonstrated that the placebo effect is a real phenomenon [1,4,5]. While it is not a miraculous cure for diseases, it can alleviate a wide number of symptoms, such as pain [6], anxiety [7], bradykinesia [8], and improve the physical [9] and cognitive [10] performance of completely healthy individuals. This is achieved through interventions that have different degrees of complexity, which can go from the white coat of a doctor—which provides the patient a feeling of trust and positive expectations [10,11]—to the administration of inactive drugs [12] and the performance of ancient rituals [13].

The effects of placebos have been hypothesized as caused by the integration of psychological, neurological, endocrine, and immune changes, which overall favor the well-being of individuals and could go hand in hand with the conventional treatment of multiple diseases [5], which is the axis of its clinical importance. Therefore, the goal of this narrative review is to describe the psycho-neuro-endocrine-immune (PNEI) basis of the placebo effect and its possible clinical applications.

## 2. Psycho-Neuro-Endocrine-Immune Basis of the Placebo Effect

**Figure 1:**
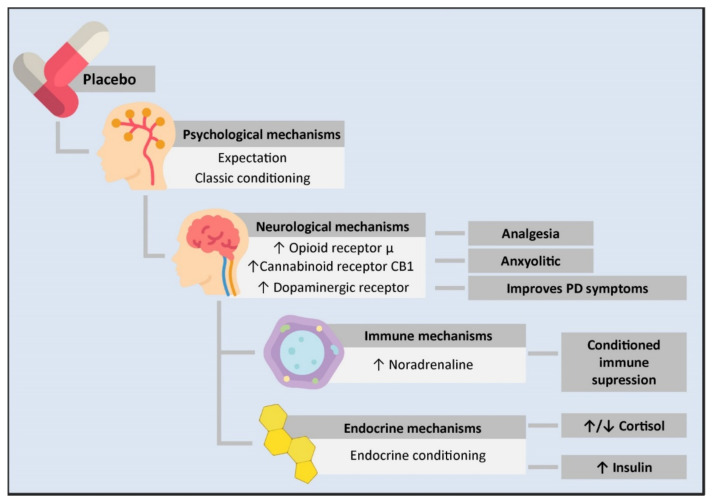
Psycho-neuro-endocrine-immune mechanisms of the placebo effect. The administration of a placebo generates a series of psychological and physiological changes in an individual. From the psychological standpoint, it can cause expectations or work as a conditioned stimulus. This is transmitted at the neurological level through an increase in the neurotransmission of μ opioid receptors in the rostral, pre and subgenual anterior cingulate cortex, the prefrontal dorsolateral cortex, the orbitofrontal cortex, the anterior insular cortex, the nucleus accumbens, the amygdala, the thalamus, and the periaqueductal substantia nigra. This mechanism is involved in placebo-mediated analgesia and anxiolytic responses. Likewise, there is an increase in the transmission of the CB1 cannabinoid receptor in placebo analgesia conditioned with non-opioid mechanisms and an increase in dopaminergic transmission in PD patients treated with placebos, leading to clinical improvement. On the other hand, the close communication between the CNS and the immune system allows for conditioned immune suppression. In this mechanism, the insulate cortex, the amygdala, the sympathetic nervous system as the main efferent pathway, and noradrenaline as the neurotransmitter responsible for immune suppression are involved. Likewise, a conditioned response in different components of the endocrine system has been observed. However, the mechanisms mediating this have not been described yet. PD: Parkinson’s disease.

During the last years, clinical use of the placebo effect has led to the development of different hypotheses regarding the molecular basis involved in its mechanism of action. Mainly, it has been discovered that is capable of generating psychological, neurobiological, immune and endocrine effects (Figure 1). In the following sections, we discuss the PNEI molecular mechanisms of the placebo effect.

### 2.1. Psychological Mechanisms: The Power of the Mind

Different psychological mechanisms are involved in the placebo effect. Among these, are expectations, learning, memory, and motivation. While there is growing research involving these mechanisms, the most studied and supported ones by experimental evidence are classic conditioning and expectations [14].

Physical conditioning is the creation of a connection between a new stimulus and an already existing reflex. Therefore, it is a type of learning in which an originally neutral stimulus causes a reaction thanks to the association of this stimulus with the one that normally leads to said response [15]. Conditioned stimuli in the placebo effect arise from the clinical setting [16,17], and the answers to the placebo are not limited to “inactive” interventions. Treatments with effective active ingredients also function as a conditioned stimulus, so in addition to having therapeutic effects based on their inherent pharmacological properties, they can generate a placebo response that improves the therapeutic benefit of the treatment [17].

It is also important to highlight the implications of the doctor–patient relationship in the psychological mechanisms of the placebo effect. It has been demonstrated that explicit verbal information can increase the efficacy of the placebo effect in conditioned analgesia [18]. On the other hand, the transference phenomenon, which is the tendency of the patient to see the doctor as an important person from their past [19], is related to classical conditioning [20] and it generates expectations.

Expectations are what the patient believes they will experience with a treatment, and it has been proven that they have a transcendental impact on what is perceived. When the patient consciously expects a positive result based on elements such as verbal instructions, previous experiences, and emotional changes, among others, this leads to internal changes that determine specific beneficial experiences [21,22]. Wanting to get better is another psychological aspect that could directly interact with expectations and amplify or reduce the placebo effect, as well as emotions. However, this has been studied to a lesser degree [23].

### 2.2. Neurobiological Mechanisms Underlying Placebo Effects

Thanks to advances in neuroimaging studies, it has been possible to identify the areas of the brain involved in the mechanisms of the placebo effect. Similarly, the neurotransmitters responsible for this phenomenon have been described, demonstrating that there are different neurobiological pathways involved in this effect [5].

The most widely described placebo effect is placebo analgesia. Therefore, this type of placebo has been used as the main model to describe the neurobiological mechanisms involved in this phenomenon. When a painful stimulus is received, the nociceptive signal ascends through the nervous fibers of the spinal cord, reaching different parts of the encephalon such as the periaqueductal grey substance, the hypothalamus, the thalamic nuclei, the amygdala, and the rostral anterior cingulate cortex [24]. In individuals responsive to placebos, the activity of these regions was reduced with the administration of a placebo, and an increase in functional connectivity between the rostral anterior cingulate cortex and the brain stem was observed [25,26,27]. Similarly, due to the expectation caused by the context of the placebo, an increase in the activity of the prefrontal cortex and the nucleus accumbens was observed [5,25,27,28]. These areas play a fundamental role in the integration of emotion/motivation, cognition, reward, and learning [29,30]. Therefore, they are instrumental for the placebo effect and it has even been reported that temporary interruption of the functionality of the prefrontal cortex through magnetic transcranial stimulation can completely block placebo analgesia [31].

These circuits are involved in affective and motivational states; therefore, the anti-anxiety and antidepressant effects of placebos are mediated by the same brain regions involved in analgesia. Mainly, these include the amygdala, the orbitofrontal cortex, and the dorsal cingulate cortex [32,33], which have important roles in the development of emotions, memory, and fear management, among others.

#### 2.2.1. Role of Opioids

In the context of neurotransmitters, placebo analgesia is mediated mainly by the endogenous opioid system. Opioids are a group of drugs that have been heavily used throughout history for analgesia purposes [34,35]. In 1975, encephalins were discovered, becoming the first endogenous opioid peptides [36]. Since then, β-endorphins [37], endomorphins [38], and dynorphins [39] have been discovered. All these are endogenous ligands of opioid receptors, which are transmembrane proteins coupled to G proteins divided into three groups: mu (μ) [40], delta (δ) [41], and kappa (κ) opioid receptors [42]. These are distributed in the peripheral and central nervous system (CNS) [42], in immune cells such as lymphocytes and macrophages, in the suprarenal glands, the heart, the liver, the lungs, and the kidneys [43].

The type of receptor (μ, δ, κ) leads to a different response once an opioid couples to it, with the α subunit of the G protein exchanging its GDP molecule for GTP. The α-GTP and βγ subunits dissociate and interact with target proteins. Classic opioid agonists lead to the inhibition of adenylate cyclase, causing a decrease in the levels of cAMP, an increase in K^+^ conduction, and a decrease in Ca^++^ conduction. This causes the hyperpolarization of the cell and, in the case of neurons, a decrease in neurotransmitter secretion [44].

Likewise, opioids lead to a decrease in both the neuronal excitation of the dorsal root ganglia of the spinal cord as well as the excitatory postsynaptic currents produced by glutamate in the spinal cord. This results in a reduction in the transmission of nociceptive stimuli, and therefore, decreased perception of pain [45].

The first evidence of the involvement of the opioid system in the placebo effect emerged from a study performed by Levine et al. in 1978. It included a sample of 27 men and 24 women aged between late adolescence and 30 years of age who underwent extraction of the third mandibular molar. It was concluded that the administration of antagonists of opioid receptors μ can inhibit placebo analgesia in post-operatory pain [46,47]. Later studies showed an increase in β-endorphins in the cerebrospinal fluid (CSF) of patients responsive to placebos [48] and demonstrated that placebo analgesia is mediated exclusively by μ, as selective inhibitors of δ and κ receptors did not decrease the placebo effect [49]. In addition, genetic analyses suggest that the polymorphism of the mu opioid receptor gene (*OPRM1*) are directly involved in the interindividual variation seen in placebo analgesia [50].

Likewise, the use of positron emission tomography (PET) with [11C]carfentanyl as a marker has led to discovering which CNS regions show an increase in neurotransmission mediated by μ opioid receptors caused by the administration of placebo with analgesia expectations. Among these regions are the pre- and subgenual rostral anterior cingulate cortex [47,51,52,53,54], the dorsolateral prefrontal cortex [51,53,54], the orbitofrontal cortex [52,54], the anterior insular cortex [51,52,53,54], the nucleus accumbens [51,52,53], the amygdala [52,53,54], the thalamus [53,54], and the grey periaqueductal substance [52,53]. These regions of the brain are associated with pain modulation, emotions, and the brain reward system. Furthermore, studies have shown spinal nociception inhibition as part of placebo analgesia [55].

Although the antinociceptive opioid system is the most widely documented, it is not the only one. It has been observed that despite the administration of naloxone, an antagonist of opioid receptors, placebo analgesia can still exist in certain circumstances [56]. This has been shown in an experimental study performed by Vase et al. in 2005 in a sample of 26 female patients with irritable bowel syndrome (IBS). These were divided into two groups, with one group receiving saline IV solution and the other one receiving naloxone. At the same time, they received rectal lidocaine (RL), rectal placebo (RP), or no intervention (natural history, NH) during three sessions that took place on different days. The results showed that, compared with NH, pain classifications were significantly lower with the administration of RL or RP. However, there was no significant difference between RL and RP. Similarly, the result was the same in the group with saline solution and the naloxone group, suggesting that the placebo effect was not mediated by opioids in this case [57].

This sets the basis for the hypothesis that different stimuli can cause different types of placebo effect, and that analgesia placebo can be produced by opioid and non-opioid mechanisms [16,58]. These last ones have not been as widely studied; however, evidence suggests that they are mainly mediated by the endocannabinoid [56] and dopaminergic systems [59].

#### 2.2.2. Endocannabinoid System

The elements that form the endocannabinoid system are cannabinoid (CB) receptors coupled to protein G, which are CB1—particularly abundant in the brain cortex, amygdala, basal ganglia, hippocampus, and cerebellum [60]—and CB2 which is mainly present in the microglia and vascular elements [61]; its endogenous ligands, which are anandamide (arachidonoyl ethanolamide) and 2-arachidonoyl glycerol; the enzymes that synthesize these ligands (Phospholipase D, specific to N-acylphosphatidylethanolamine, and diacylglycerol lipase α); and degrading enzymes (fatty acid amide hydrolase (FAHH) and monoacylglycerol lipase). These components are present in the periphery as well as central areas of the nociceptive pathway. Endocannabinoids have antinociceptive effects in neuronal circuits through the retrograde transmission of CB1 presynaptic transmitters [62].

The participation of endocannabinoids in placebo analgesia has been demonstrated when non-opioid drugs are administered and these are later replaced by a placebo and when antinociceptive effects are not completely reversible with naloxone [56,58]. Furthermore, the CB1 receptor antagonist, rimonabant, completely suppresses placebo analgesia [63]. Likewise, it has been proven that homozygotes *FAAH Pro129/Pro129*, which is the FAAH coding gene for the first endocannabinoid-degrading enzyme, resulted in higher placebo analgesia effects and mediate more positive affective states as well during the 24 h after the administration of the placebo [64]. An explanation for this could be that high concentrations of endocannabinoids cause desensitization of the CB1 receptor [65].

#### 2.2.3. Dopaminergic System

The dopaminergic system is formed by dopamine, a catecholaminergic neurotransmitter mainly synthesized in the central nervous system, especially in the substantia nigra, and the dopamine receptors. These differentiate into five subtypes (D1, D2, D3, D4, and D5) and they are coupled to G proteins. The receptors mediate all dopamine physiological functions. Among these functions are motor regulation, motivation, excitation, reward, cognitive function, pleasure, sexual behavior, breastfeeding, and nausea [66].

Dopamine cannot be classified only as an excitatory or inhibitory neurotransmitter as this will depend on the intervening receptor, second messenger response, the activation of the ion channel on the postsynaptic plasmatic membrane, and protein expression profiles [67]. Dopamine receptors are classified into type D1 receptors (D1 and D5), which are coupled to Gαs proteins that stimulate the production of cAMP through the activation of the adenyl cyclase enzyme; and type D2 receptors (D2, D3, and D4), which are coupled to Gαi, which inhibits cAMP production. In addition to cAMP regulation, dopamine receptors can have biological effects through alternative signaling pathways, such as regulating calcium channels and Na^+^/K^+^ ATPase through direct protein–protein interaction [68].

Different authors have linked the mesolimbic system, which is one of the main dopaminergic circuits, to placebo analgesia, mainly in reward anticipation. Dopamine release can mediate the analgesic effects of some placebos by decreasing activity in regions sensitive to pain, including the thalamus, insula, the anterior cingulate cortex [11,69,70], and the ventrolateral prefrontal cortex [71].

An important aspect of dopamine is that it can participate in different placebo effects, not only analgesia, as its mechanism of action is related to expectations. Therefore, it can intervene in a great number of conditions. For example, the expectation of caffeine consumption causes dopaminergic placebo effects similar to those seen in people who have received oral caffeine [72]. Most studies evaluating the implications of dopaminergic pathways are not focused on analgesia, but on the improvement of clinical symptoms caused by placebo effects in patients with Parkinson’s Disease (PD).

Motor difficulty in PD patients is caused by a decrease in dopamine release by dopaminergic neurons in the substantia nigra and the striatum as well as by the death of these neurons [73]. The benefits of the placebo effect in PD arise from the activation of the damaged nigrostriatal dopaminergic system as well as the mesolimbic pathway. PET studies used the coupling of [11 C] raclopride (RAC) to the D2/D3 dopamine receptors as an index of dopamine activity, showing that the administration of placebo in PD patients increased the release of dopamine in the dorsal striatum (caudate and putamen nuclei), especially in those responsive to placebos. This was also observed in the ventral striatum (accumbens nucleus), although no differences were observed between subjects responsive to placebos and those who were not responsive. Therefore, it was concluded that dopamine release in the ventral striatum is due to reward expectation and not the reward itself [74,75,76].

Likewise, dopamine release was larger in patients that were told they had a 75% chance of receiving the real treatment compared with those that were informed of a lower percentage (25%, 50%), and even those informed of a larger percentage (100%). Therefore, there is a possibility that uncertainty also plays a role in dopamine release caused by the placebo effect [77,78]. Different researchers have reported that placebos could possibly generate addiction in individuals who consume them, although the mechanisms that lead to dependence remain unknown [79,80].

In opposition to the previously described mechanisms, cholecystokinins (CCK) are inhibited by the placebo effect, and even proglumide, an antagonist of CCK-1 and CCK-2 receptors, can potentiate the placebo effect. It can also inhibit nocebo hyperalgesia, showing that CCK acts as a counterpart to the opioid system [16].

#### 2.2.4. Other Neurotransmitters

The aforementioned neurotransmitters might not be the only ones involved in the placebo effect. A double-blind experimental study performed by Kessner et al. in 2013 evaluated a sample of 80 healthy young men. They were injected with either oxytocin or saline solution before applying an inactive cream with expectations of analgesia. It was observed that the administration of oxytocin could improve placebo analgesia. It was hypothesized that this result was due to the effects of trust and empathy induction by oxytocin, leading to an increase in credibility regarding the instructions provided by the medical personnel to the participants [81]. However, a more recent study performed by Skvortsova et al. in 2018 with a sample of 108 healthy women between 18 and 35 years of age contradicted this discovery. There was no effect reported for oxytocin in the context of the placebo effect [82]. The greater limitation of these studies is that they did not have a representative population sample, being exclusively centered on the effect of oxytocin in just one sex.

An experimental study performed by Colloca et al. in 2016 with a sample of 109 individuals (59 women and 50 men) associated the administration of vasopressin with an increase in placebo analgesia, although this finding was observed mainly in women [83]. This study did not find an association between oxytocin and the placebo effect, but researchers point out that this could be caused by the doses of oxytocin administered, which were lower than those used in the research performed by Kessner et al. (24 vs. 41 IU, respectively) [83]. In both cases, research has been scarce and the results have been contradictory. Therefore, the participation of these hormones in the placebo effect is not entirely understood.

### 2.3. Immunological Mechanisms

It is currently known that important communication takes place between the nervous and the immune systems [84]. An example of this is the regulation of the immune response at a cellular and humoral level, mediated by neurological phenomena such as stress [85,86,87]. Likewise, it has been reported that the activation of the immune system can modify neurologic characteristics, such as mood, behavior, and anxiety levels [88].

**Figure 2:**
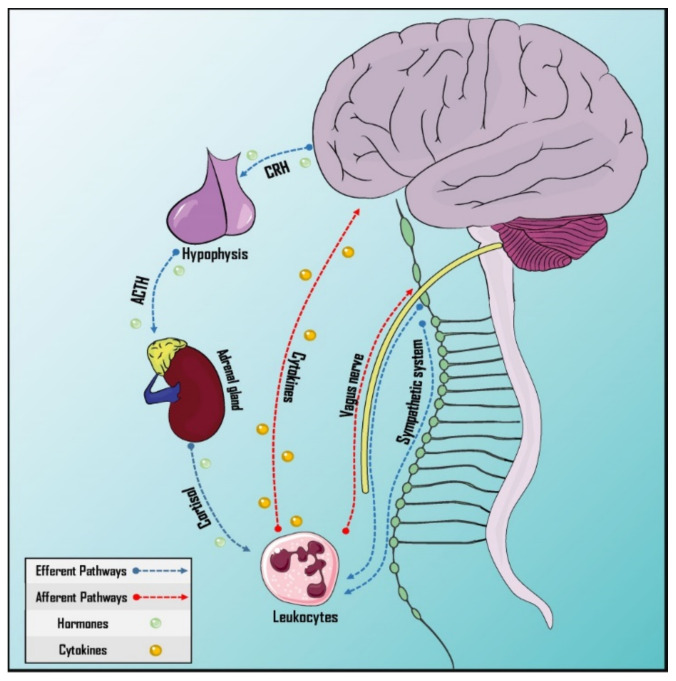
Pathways of neurommunological integration. Afferent and efferent pathways have a neural and a humoral component. The sympathetic nervous system innervates the primary and secondary lymphoid organs. The vagus nerve, which is part of the parasympathetic autonomic nervous system, has both afferent and efferent neurons, participating in both pathways. Cytokines can cross the blood–brain barrier and the hypothalamus–hypophysis–adrenal axis is responsible for humoral efference. ACTH: Adrenocorticotropic hormone; CRH: Corticotropin-releasing hormone.

Afferent and efferent pathways in this interaction are formed by neuronal and humoral mechanisms (Figure 2). In the case of the efferent pathways, the neural component is represented by the vagus nerve, representing the parasympathetic branch of the autonomic nervous system [89], and the sympathetic nervous system, which provides innervation to primary and secondary lymphoid organs and releases catecholamines, with receptors expressed by leucocytes [90]. Likewise, the humoral factor could be represented by the Hypothalamus–Pituitary–Adrenal (HPA) axis through the release of cortisol [87]. On the other hand, afferent pathways would have the vagus nerve as the neural component [89,91] and cytokines and prostaglandins as the humoral factors as these cross the Blood–Brain Barrier (BBB) [92,93].

This interaction between the CNS and the immune system makes it possible for the placebo effect to influence immune responses. In fact, it has been reported that the administration of a placebo for pain relief can reduce the levels of interleukin (IL)-18, a proinflammatory cytokine. IL-8 decrease is mediated by the placebo-induced release of endogenous opioids induced in the left nucleus accumbens [94].

However, some studies have observed that expectation alone is not capable of generating significant immunomodulation [95], for which a conditioned immune response must exist, where pavlovian learning is used to “teach” the immune system to act in a specific way when a placebo is administered [96]. This type of placebo effect can produce conditioned immunosuppression, where the sympathetic nervous system acts as the main efferent pathway [97,98] and norepinephrine acts as the neurotransmitter responsible for immunosuppression. This is suggested by the fact that propranolol, a β-adrenergic receptor antagonist, completely reverses this immunosuppressive effect [99,100].

Besides this, the immunosuppression conditioned with cyclosporine A is mainly mediated by the insular cortex and the amygdala, which are activated by the direct action of cyclosporine A in the brain through a mechanism that is still unknown, but is different from vagal afferents [101], causing a reduction in the expression and production of mRNA for IL-2, interferon-γ (IFN-γ) and T-cell proliferation [102].

This conditioned immune response can be remembered by both animal and human models [103], which was demonstrated in 2018 by Kirchhof et al. who observed conditioned immunosuppression in a sample of 30 kidney transplant patients (24 men and 6 women). This experiment took place during three phases. First, the baseline, where cyclosporine A and tacrolimus were administered as immunosuppressants to the patients and blood was extracted to analyze immune and neuroendocrine parameters. Second, the acquisition phase, in which patients received cyclosporine A, tacrolimus, and a novel gustatory stimulus serving as conditioned stimulus. Third, the evocative phase in which the administration of the immunosuppressant drug was alternated with the placebo, administering both with the green tastebud stimulant. The results of this study showed conditioned immune suppression, reflected by the inhibition of T lymphocytes when a placebo was administered instead of cyclosporine A and tacrolimus [104]. Similar results have been observed in numerous rodent studies [105].

Different experimental studies have attempted to reproduce conditioned immunostimulation, such as the studies by Buske-Kirschbaum et al. from 1992 and 1994, in which epinephrin conditioning (non-conditioned stimulus) and a neutral sorbet sweet (conditioned stimulus) increased the activity of natural killer (NK) cells [106,107]. However, this effect was not achieved in other reports, such as the one published by Grigoleit et al., a study in which conditioning with lipopolysaccharides (LPS) was attempted (non-conditioned stimulus) and a beverage with a characteristic flavor (conditioned stimulus) was provided without observing any increase in the plasma concentrations of IL-6 and IL-10, Tumoral Necrosis Factor (TNF) α, or a significant increase in body temperature once the placebo was administered, which differed from when the individuals were exposed to LPS [108]. Despite advances in the understanding of placebo immunomodulation, the precise mechanisms involved in this phenomenon are not fully understood, highlighting the importance of continued studies in this area.

### 2.4. Endocrine Mechanisms: Placebo Effect on Hormone Secretion

Hormone secretion is a process intrinsically involved in the physiologic mechanisms of multiple organ systems. Therefore, its dysregulation is a cornerstone of endocrine disorders such as diabetes mellitus, thyroid disease, and adrenal insufficiency, among others [5,109]. Hormone secretion can be defined as an unconditioned response to different non-conditioned stimuli; likewise, stimuli that take place with the non-conditioned stimulus can be associated with hormonal responses and become a conditioned stimulus. Drug consumption and the environment of the administration of a drug are one example of this [74,88]. Therefore, the use of endocrine conditioning to control hormone levels through conduct manipulation could have important clinical implications.

The effects of the application of classic conditioning have been widely studied in the endocrine system in animal and human models [109,110,111,112]. Studies have reported diverse conditioned responses depending on the non-conditioned stimuli used. Conditioned changes in corticosterone and cortisol are among the most widely researched in animal models [111,112,113,114,115]. A large proportion of these studies show significant changes in corticosterone or cortisol levels after conditioning. Barreto et al. [111] reported feeding as a non-conditioned stimulus, which led to a conditioned cortisol increase in Nile tilapia fish.

Similar findings were reported by Ader et al. [112] in a study in which an increase in conditioned corticosterone took place after the administration of cyclophosphamide as a non-conditioned stimulus. On the other hand, Coover et al. [109] reported food as a non-conditioned stimulus that led to a conditioned decrease in corticosterone. Similar to animal models, human assays analyzed the conditioned responses to cortisol, finding contradictory results between them. Sabbioni et al. [114] and Hall et al. [113] reported a significant cortisol increase, while Benedetti et al. [110] observed a decrease in cortisol levels and Stockhorst et al. [116] did not find significant results.

After cortisol and corticosterone, the conditioned release of insulin has been the most widely described response in pre-clinical and clinical settings [116,117,118,119,120,121]. Detke et al. [117] and Roozendaal et al. [118] found a significant increase in insulin levels in mice. At the clinical level, two studies performed by Stockhorst et al. [119,120] demonstrated conditioned increases of insulin in humans, using as the non-conditioned stimuli intravenous insulin and intranasal insulin, while other reports did not find significant changes in insulin levels [119,121].

Additional studies in animal models have evaluated the release of other hormones as a response to placebo. Onaka et al. [122] and Tancin et al. [123] showed a significant increase in the release of conditioned oxytocin. Similarly, Graham et al. [124] and Golombek et al. [125] demonstrated the conditioned release of testosterone, luteinizing hormone, and melatonin in mice. However, due to the scarcity of assays evaluating these hormones, more studies are necessary to replicate and confirm these findings. Finally, there are other studies at the clinical level that have evaluated the placebo effect with certain hormones without animal models. Benedetti et al. [110] and Stockhorst et al. [119] reported significant increases in growth hormone levels through classic conditioning. On the other hand, other human studies have failed to demonstrate a conditioned release of glucagon [116,119].

Despite the findings regarding conditioned responses in different components of the endocrine system, the majority of studies have certain limitations. The most remarkable one is the fact that the majority of assays were performed in men, without considering the possible moderation of the conditioned response according to gender. Therefore, future research should consider gender specificity in endocrine responses as well as evaluate other endocrine parameters that have not been entirely explored. The main human studies of the placebo effect are summarized according to the underlying mechanism in Table 1.

## 3. Preclinical and Clinical Implications of the Placebo Effect

During the last decade, there have been significant advances in the development of preclinical evidence regarding the placebo response, with the main goal of creating reproducible animal models that would bring great advantages to this field. Among these, there are advances in molecular mechanisms involved in the placebo response as well as experimental manipulations that cannot be performed in humans for technical or ethical reasons [126].

The current discussion about ethical considerations is based on aspects related to deceptive placebos and placebos without deception [127]. The first is the one that has been more extensively prohibited according to international ethical guidelines, mainly under the policy emitted by the American Medical Association in which it is declared that “Doctors can use placebos for diagnosis or treatment only if the patient is informed and accepts its use” [128]. On the other hand, a considerable number of research studies about placebos without deception have questioned the widely shared assumption that placebos require deception to be effective. Usually, in this type of study, denominated “open-label placebo”, individuals are assigned to either a group that does not receive treatment or another group that will receive a placebo pill [129]. Furthermore, patients are informed of the fact that the pill does not have any active medication and the researchers read a script to the patient informing them about placebo response and explaining the justification for the study. Recently, this methodology has provided information about statistically significant improvements in patients with chronic lumbar pain [130], IBS [131], depression [132], and recurrent migraines [133]. It has been suggested that administering placebo medication can have beneficial effects even if it is not deceptively presented as an efficient treatment.

A new type of treatment protocol has been applied in the open-label placebo model called pharmaco-conditioning to resolve possible ethical implications. In this therapeutic regime, an open-label placebo is coupled with an active drug until the administration of the open-label placebo alone induces a conditioned placebo response. The effectiveness found in different studies [134,135] suggests that this method can be effective to maintain the therapeutic response while the secondary effects of active drugs decrease. This could be a less controversial way to incorporate placebos in the clinical setting.

Currently, the available clinical evidence regarding the placebo effect is extensive and variable, especially in the case of neurologic, psychiatric, and immune disorders. The next section summarizes the key clinical evidence regarding the impact of the placebo effect as treatment for these disorders (Table 2).

### 3.1. Neurological Disorders

The placebo effect has been reported to possibly improve various neurological disorders. In this sense, the use of classical conditioning for the induction of analgesia has been extensively studied in the last 20 years. The first attempts to materialize placebo analgesia in mice led to authors reporting that taste and olfactory stimuli coupled with morphine as a conditioned stimulus caused analgesia in mice [139,140,141]. Afterward, it was demonstrated that tactile and visual stimuli coupled with the administration of morphine as a conditioned stimulus can generate placebo analgesia in female mice [58]. Zhang et al. [49] were able to replicate the results in Sprague–Dawley male rats. On the other hand, Lee et al. [59] recently proposed a new animal model of placebo analgesia. In their study, they used a conditioning paradigm in which a neutral signal was conditioned to different pain intensities in an attempt to avoid the possible stress associated with analgesia injections during conditioning phases. The authors found that in this drug-free conditioning process, there was a decrease in the nociceptive response to heat in which animals learned to associate their conditioned space with lower exposure to heat. However, studies with a larger sample and more rigorous analyses that replicate these findings are needed to determine if it is a reliable method to cause placebo analgesia in mice.

The majority of preclinical studies have been mainly based on acute pain models, with only three published animal assays attempting to examine placebo analgesia in chronic pain [142,143,144]. First, McNabb et al. [143] evaluated placebo analgesia in female mice who received a spinal clamping of the L5 nerve to induce a condition of neuropathic pain. Contextual stimuli such as the environment, time, smell, touch, and sight were used as conditioning stimuli; however, no significant differences were found. Alternatively, Zeng et al. [144] reported the induction of pharmacologically conditioned placebo analgesia using a model of spinal nerve clamping. However, this study did not include proper control groups to separate the effects of placebo from the non-specific responses that can be caused by other factors. More recently, Akintola et al. [142] approached these limitations in a rodent model of neuropathic chronic pain, finding that chronic pain in mice could be non-responsive to placebo analgesia.

Beyond the numerous studies in animals, classic conditioning has also been proven as an analgesic in humans in different pathologies associated with pain [145,146,147,148,149]. Multiple meta-analyses from clinical studies report a weak therapeutic effect on central neuropathic pain [148] and the complex regional pain syndrome, and a moderate effect in postherpetic neuralgia [150], peripheral diabetic neuropathy [150], VIH associated pain [150], fibromyalgia [151], and migraines [146,147,152]. On the other hand, only three open-label place studies related to pain have been performed to this day [130,133,153]. Carvalho et al. [130] performed an open-label, controlled, randomized study finding that placebos presented in a positive context can be used in chronic lumbar pain. In the study, patients with at least three months of chronic lumbar pain were randomly assigned to receive two tablets of placebo taken twice a day or their usual treatment for three weeks, reporting a significant decrease in their severity of pain score (95% interval confidence: 1.0–2.0). Likewise, Kam-Hansen et al. [133] performed an open-label placebo study evaluating episodic migraines, reporting superior efficiency in individuals treated with placebos compared to those that did not receive any treatment.

The placebo effects of each treatment can be used to design therapeutic strategies that improve the clinical results of the analgesic and limit its adverse effects [154]. In this context, the placebo effect induces the release of endogenous opioids that facilitate the analgesic action of exogenous opioids; therefore, it is possible to improve the response to analgesic treatments by increasing the additional placebo effect [155]. Thus, through the development of interventions that optimize the placebo effect towards the adaptation of the CNS for pain relief, a potential progressive reduction in the administration of exogenous opioids is possible [156]. There are various possibilities for taking advantage of placebo effects in the context of pain, adapting the information on analgesic treatment, and associating its intake with a positive context [155]. Based on the above, it is to be expected that the combination of analgesic drugs and placebos would have better results in reducing pain than using each strategy separately.

Recently, it has been described that the placebo effect in humans can generate an increase in dopamine release in the dorsal and ventral striatum [74,75,76], reporting that even 50% of patients with PD have shown response to placebo characterized by significant motor manifestations [77,157,158,159,160,161]. Shetty et al. [162] reported that from 36 clinical assays included in their study, 12 reported improvement after placebo treatment with PD, with a variation in the improvement from 9% to 59%. Likewise, a double-blind study found significant improvement in the group treated with pergolide and in the placebo group [163]. Alternatively, Goetz et al. [164] performed a randomized, multicenter, placebo-controlled study in which they found that 14% of patients achieved motor function improvement while they were on placebo. Another study performed by Goetz et al. [165] involved data from 11 medical and surgical assays in patients with PD. They showed that the placebo effect can be significant, especially with surgical intervention. Regarding motor symptoms, bradykinesia is the one that has the greatest response to placebo [164,166], followed by rigidity [164], gait, and tremor [164], respectively. There was a 94% improvement in bradykinesia and 59% in gait [164]. Likewise, Bennedetti et al. [167] demonstrated that the administration of placebo-induced clinical responses as large as the one from apomorphine in rigidity.

The placebo effect has also been explored in other neurological disorders. Multiple sclerosis has an unpredictable remission–relapse pattern, making it a challenge to separate the placebo effect from the natural history of the disease in clinical assays. Despite this, different neuroimaging studies have shown a decrease in the number of injuries observed in magnetic resonance in the placebo groups [136,168,169]. In an assay performed by Jacobs et al. [136] there was an improvement in the magnetic resonance of the placebo group according to what was evaluated by the number of lesions potentiated by gadolinium. In addition, a meta-analysis was performed by Beyenburg et al. [170], which included 54 studies examining anticonvulsant drugs versus placebos in over 11,106 adults and children with refractory epilepsy. They reported that there was a small difference between anticonvulsant drugs and the placebo effect [170]. Similarly, a systematic review that included 28 clinical assays evaluating multiple anticonvulsant drugs versus placebos as refractory epilepsy treatment found a response in 18% of the patients receiving placebos [171]. These results are similar to what was reported by Guekht et al., who conducted a meta-analysis that included 27 assays evaluating anticonvulsant drugs versus placebos in adults with focal epilepsy, reporting response to placebos in 12.5% of the patients [172].

### 3.2. Psychiatric Disorders

Numerous studies have researched the placebo effect in the context of current psychiatry, especially in depression. Although antidepressants offer a clear advantage over placebos in patients with severe depression, the same is not true for those with mild depression. These patients have shown a response rate to placebos close to 50%. Often, the response rate between placebos and antidepressants cannot be differentiated [173]. Furthermore, no type of psychotherapy has consistently proven to be better than placebo [174]. It has been hypothesized that common and possibly therapeutic characteristics of psychotherapy, which include improvement expectation, support, and hope mobilization, are often provided together with placebo. Different studies have estimated that the double-blind response to placebo has 80% of the strength of double-blind antidepressant response in patients with major depressive disorder in randomized controlled assays [132]. In this sense, in an open-label, randomized placebo study, a positive difference was seen among patients with major depressive disorder treated with open-label placebo and the control group. However, the difference was not statistically significant [175].

The impact of the placebo effect on anxiety disorders has been explored. It has been reported that the placebo effect in clinical assays involving this disorder ranges from 10% to 60% [176,177,178,179,180,181]. Different randomized assays have shown that the placebo response in anxiety disorders can be relevant and long-lasting. In this sense, improvement in the placebo group in clinical trials has been stable and maintained after the use of the placebo was suspended, while the patients using anxiolytic drugs suffered relapses [182,183]. On the other hand, Faria et al. [137] performed a study in which it was shown that telling patients who had been diagnosed with social anxiety disorder (SAD) that they were being treated with an active drug doubled the efficacy and tripled the response rate.

Likewise, Sandler et al. [135] demonstrated that treatment with an open-label placebo was acceptable and efficient in the short term in the case of some children with attention-deficit and hyperactivity disorder (ADHD). In the study, the behavior of kids with ADHD remained the same when the dose of the stimulus drug with the placebo was reduced, but it deteriorated when the dose without the placebo was reduced. Alternatively, Weiss et al. [184] examined the nature of the effect of placebo medication with medical treatment in alcohol dependency. It was found that the groups receiving a placebo along with medical treatment were more likely to go to Alcoholic Anonymous meetings during the treatment (32.7% and 32% vs. 20.4%) and were less likely to withdraw from treatment (14.1% and 22.9% 553 vs. 29.3%). However, more studies are needed in psychiatric settings to confirm these findings.

### 3.3. Immunological Disorders

A great number of assays evaluating classical conditioning in different immune diseases have been performed, showing its efficacy in animal models of Systemic Lupus Erythematous (SLE) [185], rheumatoid arthritis [186], and asthma [187]. In a study involving rats with experimentally induced rheumatoid arthritis, re-exposure to a solution with saccharine and vanilla flavor that had been previously combined with cyclophosphamide resulted in a decrease in inflammatory processes [188]. Likewise, in a model in rodents with SLE, mice with conditioned behavior showed relatively prolonged latency and survival time when compared to the control group animals [189].

Numerous studies have highlighted the effects of placebo response in allergies, which seems to be mediated by cognitive factors such as expectations. A decrease in symptoms of type 1 allergic reactions in people treated with placebo with previous conditioning has been reported [190]. Similarly, Goeber et al. [138] reported a placebo response in patients with allergic rhinitis. These individuals were exposed to a conditioning protocol, receiving desloratadine and a beverage for 5 days. Afterward, the patients were exposed to the beverage and a placebo, showing improvement in the symptoms after this last exposure. Different assays have also been able to show that placebo responses imitate the effects of a drug to which the subjects have been previously exposed [190,191,192]. A randomized, open-label placebo study evaluated two groups of 25 patients with allergic rhinitis comparing the use of the open-label placebo with their usual treatment for two weeks. It was observed that, at two weeks, there was a significant effect on the subjective experiences of 11 physical symptoms with significant improvement in subjective well-being (*p* = 0.009). In addition, a statistically significant reduction in symptoms was observed in the open-label placebo group when compared with the group receiving their usual treatment [193].

Likewise, different studies have reported that placebo administration leads to an improvement in objective parameters of lung function in asthma patients. These include the forced expiratory volume in 1 s (FEV1), bronchial hyperactivity, and peak expiratory flow (PEF) [187,188,194,195]. A second study found that the administration of placebowith an inhaler was beneficial according to self-reported results, with an effect similar to that of albuterol without the need for conditioning. However, no increase in FEV1 was observed in asthmatic patients treated with placebo, unlike patients treated with albuterol [196].

## 4. Conclusions

There is sufficient evidence supporting the PNEI processes involved in the placebo effect and its possible therapeutic efficacy in different diseases. Classical conditioning stands out among the psychologic mechanisms involved in the placebo effect. Neurobiological effects have been found in the opioid, cannabinoid, and monoaminergic systems, as well as possible immunomodulating responses and the secretion of hormones in both animal models and humans. At the clinical level, the epicenter of research in the last decades has been its use in pain management. However, recent studies have extended the research to include neurological, immune, and psychiatric disorders. That said, more research is needed to characterize the clinical usefulness of the placebo response in these scenarios, without forgetting the ethical concerns involved in this therapeutic option that shows the interaction of different body systems.

## Figures and Tables

**Table 1:** Psycho-neuro-endocrine-immune mechanisms of the placebo effect.

Authors (REF)	Mechanism	Methodology	Results
Carlino et al. [18]	Psychological	Thirty-four healthy subjects were divided into two groups. Both received stimulation with a CO_2_ laser and a conditioning procedure through which two visual signals were coupled with increases and decreases in the strength of the stimulus. However, one group was verbally informed about the meaning of the keys and the second group was blinded. The LEP was used as the response index.	A decrease in pain classification (*p* < 0.05) and the amplitude of LEP (*p* < 0.01) in response to the signals was reported only in the group that received verbal information. This suggests that the visual–analgesic association does not happen without explicit verbal information. This highlights the importance of cognitive content as context to the keys.
Levine et al. [46]	Neurobiological (Opioids)	A double-blind study included 51 patients who underwent extraction of their third molar. The patients were distributed in groups to administer morphine, naloxone, or placebo in a different order. Two pain classification scales were used. One was visual and the second was verbal.	When the placebo group was divided into responsive and non-responsive, it was observed that naloxone administration increased pain scores only in responders. This suggests that the analgesic effect of placebo is reversible with naloxone as this is an antagonist of μ opioid receptors.
Benedetti et al. [63]	Neurobiological (Endocannabinoids)	The effects of rimonabant (CB1 receptor antagonist) as an inhibitor of the placebo effect were evaluated in 82 volunteers, who underwent a pain test with a tourniquet for five consecutive dates. Patients were divided into six groups to receive different combinations of placebo, pain killers, and rimonabant.	It was observed that in the group who received initial placebo analgesia, rimonabant blocked the placebo response entirely. Likewise, a statistically significant difference was observed for pain tolerance in the placebo group with rimonabant and the placebo without rimonabant (*p* < 0.001).
De la Fuente-Fernández et al. [74]	Neurobiological (Dopamine)	The release of endogenous dopamine caused by the placebo effect in patients with PD was measured through PET. It was estimated according to the competition between endogenous dopamine and RAC to couple to D2-D3 receptors.	The results of the patients before and after receiving the placebo were compared. A significant decrease was found in the coupling potential of striatum RAC of 17% in the caudate nucleus and 19% in the putamen (*p* < 0.005 for both).
Kirchhof et al. [104]	Immunological	Immunologic functions and basal neuroendocrine parameters were analyzed in 30 patients. They had all had kidney transplants and had been prescribed immune suppressant drugs. Afterward, the drug was administered together with a gustative CS (acquisitive) and finally, some doses were given with a placebo and the gustative CS (evocative).	On day two of the evocative phase, decreases in the proliferative capacity of T cells (*p* < 0.001), in the expression of γ-IFN mRNA (*p* = 0.05) and of cortisol circadian plasma levels (*p* < 0.05) were reported. However, there were no changes in the number of T cells or in the levels of IL-2 and catecholamines.
Sabbioni et al. [114]	Endocrine	In a double-blind study, 25 healthy men were divided into two groups. During the first phase, one group received placebo capsules (control) while the second one received a beverage with a distinct flavor and dexamethasone capsules (experimental). During the second phase, all subjects received a beverage and a placebo capsule every other day.	It was observed that the experimental group had significantly higher cortisol plasma levels when they received the beverage and the placebo capsule (*p* = 0.015).
Stockhorst et al. [120]	Endocrine	A double-blind assay evaluated 32 healthy men who were divided into two groups. On day 1, the first group received intranasal insulin together with an olfactory CS while the second group received a placebo together with the CS. On day 2, both groups received a placebo and CS. The evaluated variables were glycemia and insulin levels.	On day 2, the levels of peripheric insulin increased in group 1 and decreased in group 2 (*p* = 0.027). While the glycemic levels decreased in group 1 and increased in group 2, there was no statistically significant difference (*p* = 0.150).

Abbreviations: LEP: N2-P2 component of laser-evoked potentials; PET: Positron emission tomography; RAC: [11C] racloprida; CS: Conditioned stimulus; UCS: unconditioned stimulus.

**Table 2:** Clinical evidence of the placebo effect.

Authors (REF)	Disorder	Methodology	Results
Carvalho et al. [130]	Neurological (Pain)	A randomized, controlled, open-label study included 83 patients who had chronic lumbar pain for less than three months. They randomly received either two tablets of placebo a day or their usual treatment for three weeks. The intensity of the pain was measured through three scales and a total score was obtained.	Greater pain reduction was observed in each of the scales in the patients who received a placebo in comparison to those receiving their usual treatment (*p* < 0.001). Similarly, those treated with a placebo had a reduction in their disability compared to those in their regular treatment (*p* < 0.001).
Jacobs et al. [136]	Neurological (Multiple sclerosis)	A Phase III, randomized, placebo-controlled, double-blind trial evaluating the use of beta-1a interferon included 301 patients with multiple sclerosis. Time to progression of disability was the primary endpoint, measured using the Expanded Disability State Scale from Kurtzke.	The placebo significantly delayed the time to progression (*p* = 0.02). Likewise, patients had fewer exacerbations (*p* = 0.03) and fewer and smaller brain lesions in magnetic resonance imaging (MRI) (*p* = 0.02).
Kelley et al. [132]	Psychiatric (Depression)	An open-label, placebo-controlled study included outpatient individuals who were diagnosed with major depressive disorder. They were randomized into a control group and an open-label placebo group.	There were no statistically significant differences between groups (*p* = 0.26). However, improvement before and after four weeks of receiving the placebo was observed (*p* = 0.03).
Faria et al. [137]	Psychiatric (Anxiety)	A trial evaluated 46 patients diagnosed with social anxiety, randomizing them to receive nine weeks of open or blinded treatment with escitalopram. The efficacy of the treatment was evaluated with the LSAS-SR and brain activity measured through MRI.	The results according to the LSAS-SR were better in the open-label group than the blinded group (*p* < 0.0001), with a response rate three times higher (50% vs. 14%; *p* = 0.009). This was correlated with greater activity of the posterior cingulate gyrus (*p* = 0.0006).
Goebel et al. [138]	Immunological (Allergic rhinitis)	Thirty patients with allergic rhinitis received a beverage with a distinct flavor followed by a dose of desloratadine for five consecutive days. Afterward, 10 patients received water together with a placebo pill (water group), 11 patients were exposed again to the beverage and the placebo pill (placebo group) and nine patients received water and desloratadine (drug group).	The water group had a decrease in symptoms and results of the cutaneous test but not in basophil activation. The placebo group had a decrease in basophils, decreased response in the cutaneous test and symptoms similar to those observed in the drug group.

Abbreviations: LSAS-SR: self-rated Liebowitz Social Anxiety Scale.

## Data Availability

Not applicable.
